# Effects of Dietary Squalene Supplementation on the Growth Performance and Disease Resistance of Largemouth Bass

**DOI:** 10.3390/vetsci13050448

**Published:** 2026-05-01

**Authors:** Shan Liu, Mengmeng Chen, Yan Meng, Mingyang Xue, Yong Zhou, Liping Zhang, Peng Chen, Yuding Fan, Yazhen Yang, Zhenyu Huang

**Affiliations:** 1 College of Life Science, Yangtze University, Jingzhou 434023, China; 2 Yangtze River Fisheries Research Institute, Chinese Academy of Fishery Sciences, Wuhan 430223, China; 3 School of Marine Biology and Fisheries, Hainan University, Haikou 570228, China; 4 Chongqing Fisheries Technical Extension Center, Chongqing 401120, China; 5 Xinjiang Uygur Autonomous Region Fisheries Development Center, Urumqi 830000, China

**Keywords:** squalene, largemouth bass, growth performance, disease resistance

## Abstract

Feed supplements play a crucial role in improving and maintaining fish health in modern aquaculture practices. Squalene is a functional lipid naturally present in fatty tissues, possessing numerous beneficial biological properties and wide applications in the food and pharmaceutical industries. In this study, the effects of 100 mg/kg (S1), 200 mg/kg (S2), 300 mg/kg (S3), and 400 mg/kg (S4) of dietary squalene supplementation over four weeks on growth performance, antioxidation, hepatoprotection, hypoxia tolerance, immune relative genes expression, and disease resistance of largemouth bass (*Micropterus salmoides*) were assessed. The results showed that squalene supplementation significantly increased the weight gain rate (WGR) and specific growth rate (SGR) of largemouth bass (*p* < 0.05). Serum glucose (GLU) levels were significantly decreased in all squalene-supplemented groups (*p* < 0.05). Squalene supplementation had minimal effect on serum triglyceride (TG) and total cholesterol in (TCHO) levels. A decrease in malondialdehyde (MDA) level, but accompanied by increases in superoxide dismutase (SOD) and hepatic catalase (CAT) activities, was observed in the S1 group supplemented with squalene. These suggest that squalene may mitigate free radical damage and promote health in largemouth bass. Dietary squalene supplementation enhanced intestinal enzyme activities (trypsin, lipase, and α-amylase) in largemouth bass without inducing any apparent hepatic or histopathological alterations. Squalene supplementation improved hypoxia tolerance and antiviral gene expression (*mx*, *ifn-γ*, and *irf3*) while suppressing the expression of inflammatory cytokine (*il-1β*, *il-8*, and *tnf-α*). The survival rate following LMBRaV infection was significantly higher in the S1 group (100 mg/kg group) compared to the control (*p* < 0.05). In conclusion, this study demonstrated that adding squalene into the diet of largemouth bass at an optimal level of 100 mg/kg effectively promotes growth performance, enhances digestive enzyme activity and hypoxia tolerance, and modulates lipid metabolism and immune gene expression, thereby contributing to improved resistance against LMBRaV. These findings confirm that squalene can serve as a beneficial functional feed additive in aquaculture.

## Simple Summary

In aquaculture, dietary supplement feed with a functional additive is an effective approach to enhancing fish health. Squalene, recognized for its diverse biological activities, has found extensive application in the food and pharmaceutical sectors. This study aimed to evaluate the effects of dietary squalene supplementation on the growth performance and health status of largemouth bass (*Micropterus salmoides*). The results demonstrated that adding squalene to the diet of largemouth bass can improve growth performance, increase serum total protein level, decrease serum glucose concentrations, and enhance digestive enzyme activity. It enhanced hypoxia tolerance, modulated immune gene expression, and improved the survival rate of largemouth bass against largemouth bass ranavirus (LMBRaV) infection. The optimal dietary supplementation level of squalene for improving growth performance and disease resistance in largemouth bass was determined to be 100 mg/kg of diet. These findings highlight the potential of squalene as a valuable dietary supplement for aquaculture.

## 1. Introduction

Largemouth bass (*Micropterus salmoides*), a fish species native to North America, was introduced into China in the 1970s and has since become an economically important aquaculture species due to its rapid growth, strong adaptability, and high environmental tolerance [1,2]. It is now widely cultivated across many regions of China, with annual production reaching 938,509 tons in 2024 [3]. With the expansion of intensive aquaculture of this species, increasing attention has been focused on research and development of formulated feeds aimed at improving farming efficiency and enhancing disease resistance. To date, various feed additives, including plant extracts [4], probiotics [5], vitamins [6], amino acids [7], tannic acid [8], phytosterol [9], acidic calcium sulfate [10], and trace elements [11] have been incorporated into largemouth bass diets with relatively promising results. Among these, herbal extracts have attracted particular interest as feed additives owing to the presence of bioactive components that can enhance growth performance, immune function, disease resistance, and nutrient absorption, as well as protect internal organs [12,13,14,15,16,17,18].

Squalene is a linear polyunsaturated triterpene hydrocarbon (C_30_H_50_) and a non-steroidal compound that serves as a key precursor in cholesterol biosynthesis [19]. Squalene was first isolated from shark liver oil in 1916 and named after the shark family Squalidae [20]; it has since been found in various animals, plants, and even microorganisms [21,22]. Driven by sustainability concerns, the development of plant-derived squalene has progressed rapidly. Consequently, this has in turn led to a significant reduction in the cost of squalene. Known plant sources of squalene encompass olive (*Olea europaea* L.), Camellia oleifera Abel, amaranth [23], and others, such as monk fruit, Chinese tallow, Rabdosia, and licorice [24,25,26,27]. Extracting active substances from these plants offers several advantages, including environmental safety, a broad spectrum of active components, and reduced health risks [28,29]. Squalene is widely utilized in the food and pharmaceutical industries as a dietary supplement, vaccine adjuvant, natural antioxidant, moisturizer, and cardioprotective or antitumor agent [22,30]. Dietary squalene supplementation improves meat quality, increases weight gain, improves oxidative status, and alleviates oxidative stress and liver damage in broiler chickens [31,32]. And in aquaculture species such as Siberian sturgeon (*Acipenser baerii*), rainbow trout (*Oncorhynchus mykiss*), and Eurasian perch (*Perca fluviatilis* L.), hybrid grouper or coin-sized *Eriocheir sinensis*, squalene supplementation has also displayed the beneficial effects in aspects such as growth performance [33,34,35]. Research indicated that squalene regulates cholesterol metabolism [36,37], exhibits anticancer and detoxifying properties [38], and also influences immune function [39,40]. Squalene plays a role in mitigating isoproterenol-induced oxidative stress in rat hearts [41] and enhances oxygen-carrying capacity [42]. Exogenous squalene intake can increase high-density lipoprotein cholesterol and paraoxonase-1 levels in blood and mitigate oxidative stress damage in mice [43], whereas it may lower serum cholesterol levels [44]. A high concentration of squalene displayed inhibitory activity against bacterial and fungal infection in dry skin lesions and eczema [45]. The antioxidant effects of squalene protect cells from free radicals and reactive oxygen [46]. Squalene promotes cardiac health through anti-inflammatory effects mediated by the NF-κB and Nrf2 pathways [47]. Squalene boosted immunity in pig skin tissue by increasing the activity of catalase (CAT) and superoxide dismutase (SOD) [48]. Additionally, squalene demonstrated anti-inflammatory and antioxidative effects in Zebrafish (*Danio rerio*) with Copper Sulfate-induced inflammation [49]. Overall, squalene demonstrates considerable potential as a nutritional supplement for aquatic animals, especially in aquafeed, to enhance growth performance and adaptability.

Therefore, this study aimed to evaluate the effects of dietary squalene supplementation as a feed additive on growth performance, antioxidant capacity, and disease resistance in largemouth bass. The findings are expected to provide valuable insights for the application of squalene in aquaculture and the sustainable development of the largemouth bass industry.

## 2. Materials and Methods

### 2.1. Ethics Statement

All experimental procedures were approved by and carried out by the Animal Experiments Monitoring Committee of the Yangtze River Fisheries Research Institute and performed in accordance with relevant ethical guidelines (No. YFI2024-zhouyong-0425).

### 2.2. Experimental Fish

Largemouth bass free of specific pathogens were sourced from a local farm in Jingzhou, Hubei Province, China. Upon arrival, the fish were acclimated to laboratory conditions for two weeks in 200-liter recirculating tanks with tap water of continuous oxygenation, temperature maintained at 24 °C ± 1 °C, and fed a commercial diet. After the acclimation period, 600 apparently healthy largemouth bass (mean body weight 17.60 ± 0.31 g) were randomly assigned to five dietary treatment groups, each comprising three replicates of 40 fish per tank. The fish were reared for 4 weeks and fed with experimental diets or commercial diets three times daily. Mortality was recorded daily throughout the feeding trial, and rearing conditions were kept consistent with those during the acclimation period.

### 2.3. Experimental Diets and Procedure

The experimental diet was purchased from a commercial feed (Tongwei, Wuxi, China). Its composition contained the crude protein (50.2%), crude lipid (9.3%), crude fiber (1.2%), crude ash (14.3%), lysine (3.1%), moisture (9.3%), and total phosphorus (2.6%). And the major ingredient types were fish meal, soybean meal, flour, vitamins, and minerals. Analysis of the basal diet confirmed that the endogenous squalene concentration was negligible. Squalene, a colorless oil with a 98% purity, was bought from a biotechnology company (Yuanye, Shanghai, China). Based on squalene supplementation levels reported in previous studies [31,33,50,51], five experimental diets were formulated with graded squalene levels of 0, 100, 200, 300, and 400 mg/kg feed. The squalene was dissolved in anhydrous ethanol and mixed thoroughly with commercial feed pellets. Then the mixture was stirred gently at room temperature (25 ± 1 °C) to naturally dry in a ventilated area to remove ethanol. The prepared experimental diet of floating pellets about 3.5 mm (mm) was then left to dry naturally. These experimental diets were named group C (control, 0 mg/kg squalene), S1 (100 mg/kg squalene), S2 (200 mg/kg squalene), S3 (300 mg/kg squalene), and S4 (400 mg/kg squalene).

### 2.4. Sample Collection and Growth Performance Measurement

All the largemouth bass in one tank were bulk-weighed at the beginning of the experiment trial. And following a 24 h fast at the end of the four-week feeding experiment, they were weighed and tallied again. Six fish were randomly selected from each tank and anesthetized with MS-222 (Yuanye, Shanghai, China) to measure standard body length, visceral weight, liver weight, and total length. These data were used to calculate the parameters, including weight gain rate (WGR), specific growth rate (SGR), survival rate (SR), condition factor (CF), viscerosomatic index (VSI), and hepatosomatic index (HSI). The indices were calculated using previously established formulas [52] as follows:

Weight Gain Rate (WGR, %) = (Final body weight − Initial body weight)/Initial body weight × 100%.

Specific Growth Rate (SGR, %/d) = [ln (Final body weight) − ln (Initial body weight)]/Experimental days number × 100%.

Survival Rate (SR, %) = (Final number of the experimental fishes/Initial number of the experimental fishes) × 100%.

Condition Factor (CF, g/cm^3^) = (Final body weight/Final body length^3^) × 100 Viscerosomatic Index (VSI, %) = (Visceral mass/body mass) × 100

Hepatosomatic Index (HSI, %) = (Final liver weight/Final body weight) × 100.

Blood samples were collected from the caudal vein of six fish using sterile syringes. The blood from each fish was loaded into a 2 mL sterile centrifuge tube and centrifuged at 1000× *g* for 10 min. The supernatant (serum) was collected for subsequent biochemical analysis. After blood collection, liver, spleen, and intestine were immediately excised from the same fish, snap-frozen on dry ice, and stored at −80 °C for subsequent analysis.

### 2.5. Serological Analysis

Serum biochemical parameters were analyzed by the BX-4000 automated biochemical analyzer (Sysmex, Tokyo, Japan). The measured liver function-related parameters included total protein (TP), albumin (ALB), aspartate aminotransferase (AST), total bilirubin (TBIL), and alkaline phosphatase (ALP). Lipid metabolism-related parameters include triglycerides (TG) and total cholesterol (TCHO). In addition, the serum glucose (GLU) levels were determined. All assays were performed in duplicate using samples from three individual fish per tank.

### 2.6. Antioxidant and Digestive Activity Analysis

Liver samples were analyzed for the antioxidant activities based on the antioxidant enzyme of superoxide dismutase (SOD) and hepatic catalase (CAT), as well as malondialdehyde (MDA), which is a core indicator for assessing the status of oxidative metabolism. Also, Intestinal samples were analyzed for the activity of digestive enzymes, including trypsin, lipase, and α-amylase (AMS). Liver and intestine samples stored at −80 °C were thawed at 4 °C. Residual blood or moisture was removed using filter paper, and the tissues were then thoroughly homogenized in ice-cold physiological saline. After centrifugation at 3000 rpm for 10 min at 4 °C, the protein concentration of the supernatant was determined using the BCA method. All enzyme assays were performed using commercial kits (Nanjing Jiancheng Bioengineering Institute, Nanjing, China) following the manufacturer’s instructions. Each assay was conducted in duplicate using samples from three individual fish per tank.

### 2.7. Histomorphological Observation

Three largemouth bass from each tank were dissected to collect the liver and intestine tissues from the same site of each fish. The tissues were fixed in 4% paraformaldehyde for 24 h at room temperature, then embedded in paraffin, sectioned at 5 μm, and stained with hematoxylin and eosin (H&E). Histomorphological changes in the liver and intestine were observed and imaged using an Olympus BX53 light microscope (Olympus, Tokyo, Japan).

### 2.8. Hypoxia Tolerance Test

After the 4-week feeding trial, six largemouth bass from each tank were randomly selected and placed into sealed fish bags filled with water. The dissolved oxygen concentration in water, the time, and the death situation of fish of each group were monitored and recorded. The dissolved oxygen concentrations at the beginning of the fish bag, at the fish semi-lethal point, and at the fish total lethal point were all measured, respectively, by a dissolved oxygen analyzer (Octadem, Wuxi, China). The difference between the oxygen concentration at the semi-lethal point or total lethal point and the initial concentration was used to assess the tolerance to oxygen.

### 2.9. Immune-Related Genes Expression

Spleen samples were collected from each group on the 14th and 28th days of the feeding trial and stored in TRIzol reagent (Invitrogen, Carlsbad, CA, USA) for RNA extraction. Total RNA was isolated and reverse transcribed into cDNA using a reverse transcription kit (Yisheng, Shanghai, China) following the manufacturer’s instructions. Six immune-related genes, including *mx* (myxovirus resistance), *ifn-γ* (interferon gamma), *irf3* (interferon regulatory factor 3), *tnf-α* (tumor necrosis factor-alpha), *il-8* (interleukin-8), and *il-1β* (interleukin-1beta), were selected to evaluate the effects of dietary squalene on immune function in largemouth bass. RT-qPCR analysis was carried out using gene-specific primers (listed in Table 1), with *β-actin* serving as the internal reference gene. Relative gene expression levels were calculated using the 2^−ΔΔCt^ method [53]. All reactions were performed in triplicate.

### 2.10. LMBRaV Challenge Experiment

After the 28-day feeding trial, 20 fish from each group were selected for viral challenge with LMBRaV. The LMBRaV strain used here was maintained in our laboratory. Each fish was intraperitoneally injected with 100 µL of the diluted LMBRaV suspension (10^4^ TCID_50_/mL). Following the LMBRaV challenge, mortality was recorded daily for 14 days. Finally, the cumulative survival rate was calculated for each group.

### 2.11. Statistical Analysis

All data were analyzed using GraphPad Prism version 8.0. Differences among groups were assessed by one-way analysis of variance (ANOVA), and results were assessed as the mean ± standard deviation (SD). Survival curves were generated using the Kaplan–Meier method. Differences between treatment groups and control groups were determined using Dunnett’s test. Statistical significance was set at ** indicated *p* < 0.01 and * indicated *p* < 0.05.

## 3. Results

### 3.1. Effect of Squalene on Growth Performance

The effects are presented in Table 2. All squalene supplementation groups except the S4 group exhibited higher WGR and SGR compared to the control group. Among the squalene-supplemented groups, the S4 group (400 mg/kg) displayed lower WGR and SGR than the other three treatment groups. SR was significantly higher in the S1 and S2 groups than in the control group (*p* < 0.05), whereas the S3 and S4 groups had lower SR than the control group. No differences were observed in condition factor (CF) among any of the groups. Similarly, viscerosomatic index (VSI) and hepatosomatic index (HSI) did not differ significantly between experimental groups and the control group, nor among the treatment groups themselves. The S1 group (100 mg/kg) exhibited the most favorable outcomes in terms of WGR, SGR, SR, CF, and VSI.

### 3.2. Effect of Squalene on Serum Biochemistry Parameters

**Figure 1:**
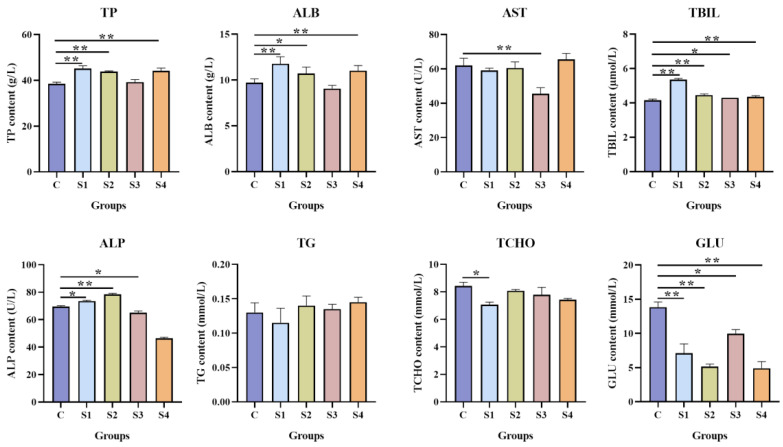
Effects of squalene supplementation on serum biochemical indices of largemouth bass. TP: total protein, ALB: albumin, TBIL: total bilirubin, ALP: alkaline phosphatase, TG: triglyceride, TCHO: total cholesterol, GLU: glucose, AST: aspartate aminotransferase. All values are expressed as mean ± SD, ** *p* < 0.01; * *p* < 0.05. *n* = 6. C (control, 0 mg/kg squalene group), S1 (100 mg/kg squalene group), S2 (200 mg/kg squalene group), S3 (300 mg/kg squalene group), S4 (400 mg/kg squalene group).

The serum biochemistry parameters related to liver function and blood lipids are presented in Figure 1. Dietary squalene supplementation affected several liver function indicators to varying degrees. The S1, S2, and S3 groups exhibited significantly higher total bilirubin (TBIL) and alkaline phosphatase (ALP) levels compared to the control group (*p* < 0.05). Total protein (TP) and albumin (ALB) levels did not differ significantly between the S3 group and the control group (*p* > 0.05). Notably, TBIL and ALP levels showed an initial increase followed by a decrease with increasing dietary squalene concentration. Squalene supplementation had minimal effects on serum triglycerides (TG) and total cholesterol (TCHO)across most treatment groups (*p* > 0.05), with the exception of the S2 group, which showed significantly lower TCHO levels than the control group (*p* < 0.05). In contrast, serum glucose (GLU) levels were substantially reduced in all squalene-supplemented groups compared to the control group (shown in Figure 1).

### 3.3. Effect of Squalene on Antioxidant and Digestive Activity

**Figure 2:**
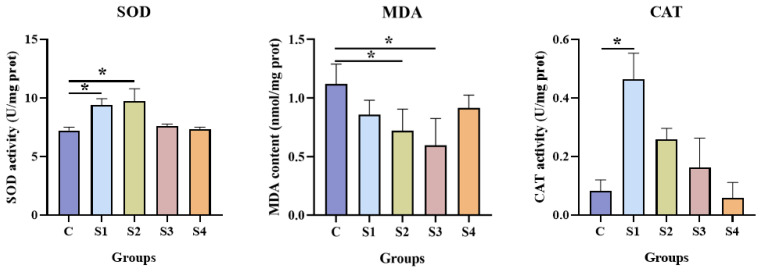
Antioxidant activity in the liver of largemouth bass following squalene supplementation. SOD: Superoxide Dismutase. MDA: malondialdehyde. CAT: catalase. Values are expressed as mean ± SD; * *p* < 0.05. *n* = 6. C (control, 0 mg/kg squalene group), S1 (100 mg/kg squalene group), S2 (200 mg/kg squalene group), S3 (300 mg/kg squalene group), S4 (400 mg/kg squalene group).

The hepatic antioxidant activity is shown in Figure 2. Compared to the control group, SOD activity was significantly increased in the S1 and S2 groups (*p* < 0.05). MDA levels were lower in the S1, S2, and S3 groups than in the control group. No significant differences in CAT activity were observed between the control group and the S2, S3, or S4 groups (*p* > 0.05); however, CAT activity in the S1 group was significantly higher than that in the control group (*p* < 0.05).

**Figure 3:**
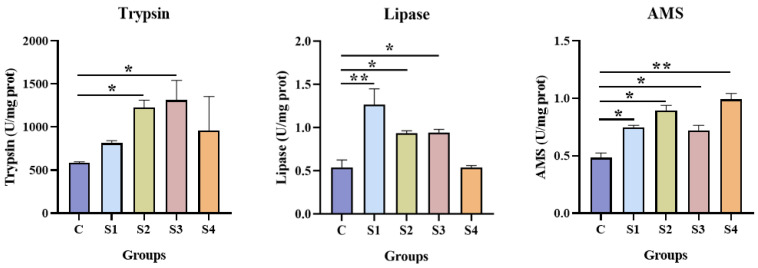
Effect of squalene supplementation on intestinal digestive enzyme activity of largemouth bass. All values are expressed as mean ± SD. ** *p* < 0.01; * *p* < 0.05. *n* = 6. C (control, 0 mg/kg squalene group), S1 (100 mg/kg squalene group), S2 (200 mg/kg squalene group), S3 (300 mg/kg squalene group), S4 (400 mg/kg squalene group).

Intestinal digestive enzyme activities are shown in Figure 3. Compared with the control group, dietary squalene supplementation increased the activities of trypsin, lipase, and AMS. The trypsin activity was higher in the S2 and S3 groups than in the control group. The lipase activity in the S4 group did not differ from that in the control group. In contrast, the AMS activity was significantly higher in all squalene-treated groups than in the control group (*p* < 0.05).

### 3.4. Effect of Squalene on Histological Change

**Figure 4:**
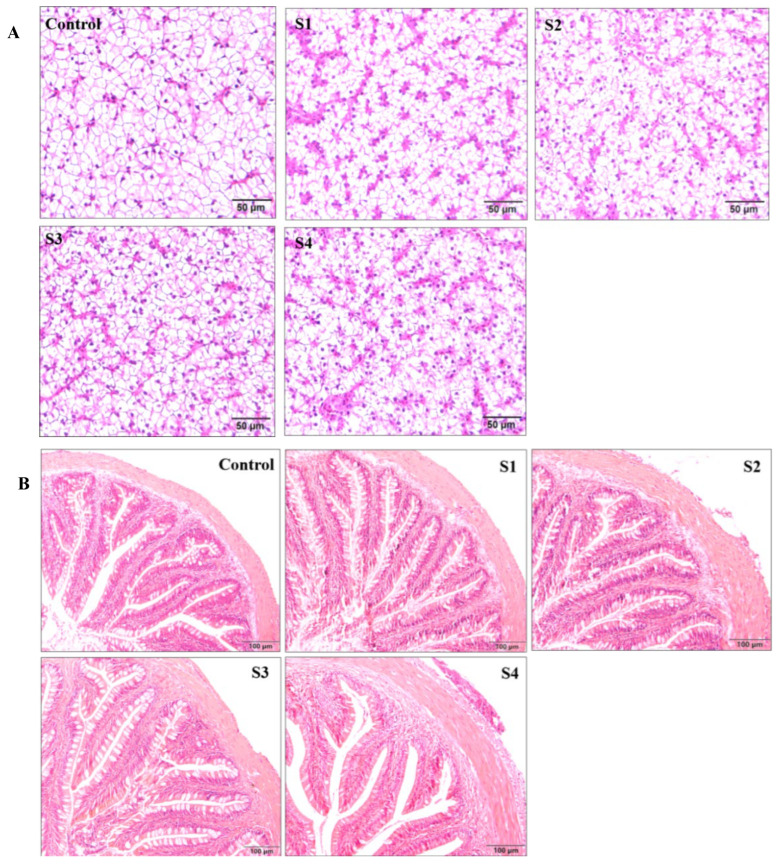
Effect of squalene on histomorphology of largemouth bass. (**A**). Liver morphology. Scale bars: 50 μm. (**B**). Intestinal morphology. Scale bars: 100 μm. *n* = 3, S1 (100 mg/kg squalene group), S2 (200 mg/kg squalene group), S3 (300 mg/kg squalene group), S4 (400 mg/kg squalene group).

Histological changes in the liver and intestine are presented in Figure 4. In all groups, the hepatocytes were orderly and neatly arranged, exhibiting normal tissue morphology. The liver cells in squalene-supplemented groups were densely packed, with no signs of cellular damage or inflammation. In the intestinal tissue, morphology was intact and complete across all groups, with clear boundaries and normal microstructure. Intestinal villi in squalene-treated groups displayed a smooth striated border. Notably, the S1, S2, and S3 groups showed better intestinal tissue morphology than the control and S4 groups, characterized by regular, well-defined villi that appeared more intact intestinal villus structures and in a more regular arrangement, potentially enhancing digestive capacity.

### 3.5. Effect of Squalene on Hypoxia Tolerance

**Figure 5:**
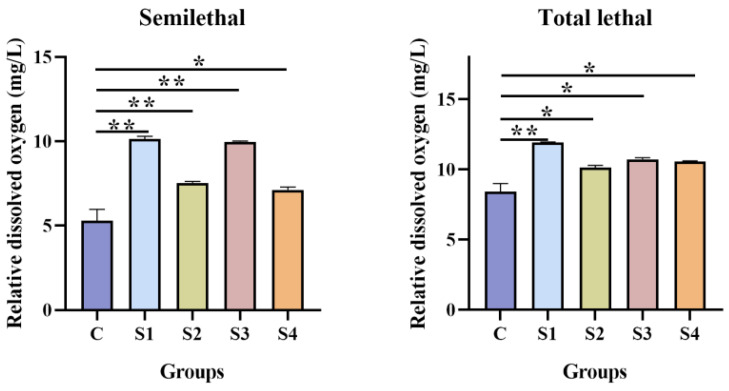
Effect of squalene on hypoxia tolerance of largemouth bass. All values are expressed as mean ± SD, ** *p* < 0.01; * *p* < 0.05. *n* = 12. C (control, 0 mg/kg squalene group), S1 (100 mg/kg squalene group), S2 (200 mg/kg squalene group), S3 (300 mg/kg squalene group), S4 (400 mg/kg squalene group).

The hypoxia tolerance test demonstrated that all squalene-supplemented groups exhibited significantly higher dissolved oxygen concentrations at both the semi-lethal and total-lethal points compared with the control group (p < 0.05; Figure 5). Notably, the S1 group (100 mg/kg squalene) showed significantly higher dissolved oxygen than the control group (*p* < 0.01).

### 3.6. Effect of Squalene on Immune Gene Expression

**Figure 6:**
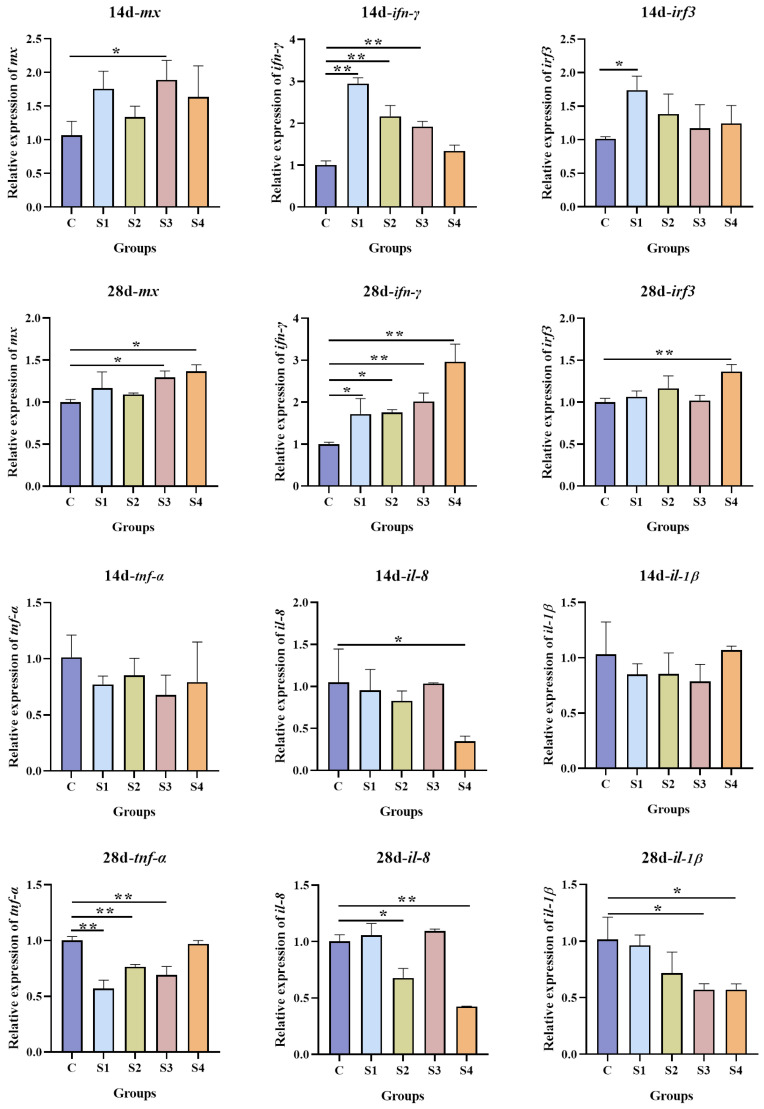
Effects of squalene supplementation on the expression of immune-related genes and inflammatory cytokine-related genes in the spleen of largemouth bass. The expression of mx, ifn-γ, irf3, tnf-α, il-8, and *il-1β* was detected by RT-qPCR. All values are expressed as mean ± SD, ** *p* < 0.01; * *p* < 0.05. *n* = 3. C (control, 0 mg/kg squalene group), S1 (100 mg/kg squalene group), S2 (200 mg/kg squalene group), S3 (300 mg/kg squalene group), S4 (400 mg/kg squalene group).

The expression of six immune-related genes is displayed in Figure 6. The antiviral genes *mx*, *ifn-γ*, and *irf3* were upregulated to varying degrees in squalene-supplemented groups. *Mx* expression was significantly higher in the S3 and S4 groups at 28 days (*p* < 0.05). The *ifn-γ* expression was significantly increased in all squalene-treated groups at day 28 (*p* < 0.05). The *irf3* expression was elevated in the S1 group at 14 days and in the S4 group at 28 days (*p* < 0.05). In contrast, the inflammatory cytokines of *il-10*, *il-1β*, and *tnf-α* tended to be downregulated by squalene supplementation. No significant differences were detected at 14 days (*p* > 0.05). In contrast, at 28 days, the *tnf-α* and *IL-8* expression were significantly reduced in the S2 group, and *tnf-α* and *il-1β* were significantly reduced in the S3 group (*p* < 0.05).

### 3.7. Effect of Squalene on LMBRaV Infection

**Figure 7:**
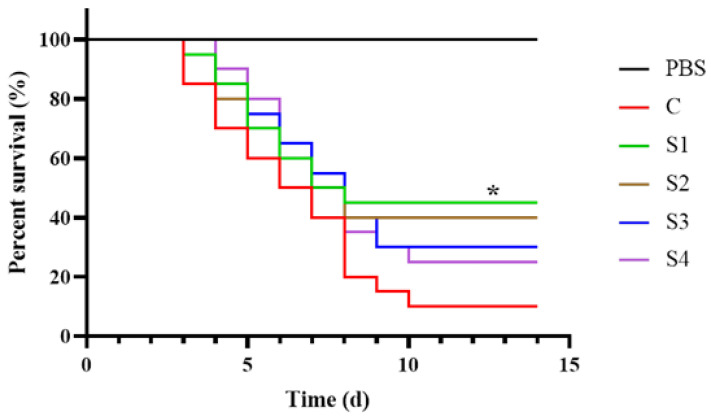
Effect of squalene on cumulative LMBRaV-infected largemouth bass, * *p* < 0.05. C (control, 0 mg/kg squalene group), S1 (100 mg/kg squalene group), S2 (200 mg/kg squalene group), S3 (300 mg/kg squalene group), S4 (400 mg/kg squalene group).

The survival rate of largemouth bass following LMBRaV-infected is illustrated in Figure 7. Compared with the control group, the S1 group exhibited a significantly higher survival rate (40% vs. 10%, *p* < 0.05). In contrast, no significant differences in survival rates were observed between the control group and the S2, S3, or S4 groups (*p* > 0.05).

## 4. Discussion

Squalene is a functional lipid found in fatty tissues and possesses numerous beneficial biological properties. It has been widely used in the food and pharmaceutical industries. In this study, we evaluated the effects of dietary squalene supplementation on the growth performance, enzyme activity, serum biochemical parameters, tissue morphology, immune response, and disease resistance in largemouth bass. The results confirmed that squalene effectively enhances both growth and disease resistance in largemouth bass.

Growth performance indicators such as specific growth rate, weight gain rate, and survival rate are crucial parameters for evaluating animal growth performance. Previous studies have suggested that squalene can enhance growth or reproductive performance in various animal species. For example, dietary squalene supplementation at 1–2 kg/tonne (equivalent to 0.1%) significantly improved average daily weight gain and feed conversion ratio in broilers [31]. Similarly, supplementation with 250 mg/kg (equivalent to 0.025%) squalene enhanced growth performance and reduced the incidence of diarrhea in weaned piglets [50]. Administering squalene to 12-month-old boars at doses of 10, 20, or 40 mg/kg (equivalent to 0.001%, 0.002%, and 0.004%) body weight per day for 60 days revealed that supplementation at 20 mg/kg or 40 mg/kg remarkably improved reproductive performance [56]. Studies on aquatic species have reported variable effects of squalene supplementation. In Siberian sturgeon, rainbow trout, and Eurasian perch, diets containing 0.5% and 1.0% squalene were found to be safe, and supplementation increased n-3 polyunsaturated fatty acids (PUFA) and docosahexaenoic acid levels in all fish groups; however, no significant growth-promoting effects were observed [34]. Adding 0.05%, 0.1%, and 0.2% squalene to the basal diet of hybrid grouper, dietary squalene levels did not significantly affect the WGR, SGR, or morphological indices of groupers, but the feed conversion ratio (FCR) of the 0.05% addition group was significantly lower than that of the other three groups [33]. In coin-sized *Eriocheir sinensis*, another aquaculture species but not a fish, dietary squalene supplementation at levels of 0.05%, 0.1%, 0.2%, and 0.4% feed significantly enhanced the final body weight (FBW), weight gain rate (WGR), and specific growth rate (SGR), with the 0.4% level providing the best growth-promoting effect [35]. In this present study, dietary squalene supplementation at levels of 100, 200, 300, and 400 mg/kg (equivalent to 0.01%, 0.02%, 0.03%, and 0.04%) in largemouth bass commercial feed exhibited varying degrees of beneficial effects on growth parameters of WGR, SGR, and CF, with the 100 mg/kg dosage showing the most pronounced improvement. Exogenous substances often exhibit dose-dependent effects in animals. However, because no intermediate concentrations between 0 and 100 mg/kg were included, the conclusion that 100 mg/kg is the optimal supplementation level is limited to the dose range tested in this study. The doses used in our study are lower than those reported in some previous studies. These results indicate that the effects of squalene supplementation on growth performance vary with dosage as well as animals, but overall remain safe and beneficial. The effects of exogenous squalene may vary among animal species due to differences in metabolic requirements and endogenous squalene levels. It is known that fish contain a relevant level of squalene, and the content varies among different species. Such variability can lead to cumulative effects that influence the overall physiological response to supplementation. However, in any case, the potential of squalene as a functional feed additive remains substantial, particularly for aquaculture species.

Hematological and biochemical analyses are fundamental methods for rapidly assessing an organism’s inflammatory state and metabolic status, thereby providing insights into an animal’s overall health [57]. Different parameters reflect distinct physiological conditions. For example, serum TP level reflects protein metabolism and nutritional status, while serum ALP is associated with phosphate group transfer and calcium-phosphorus metabolism [58]. Serum TCHO and TG levels reflect lipid metabolism, and plasma AST activity serves as an indicator of hepatocellular damage [59,60]. In this study, serum levels of TP, ALB, TBIL, ALP, TCHO, TG, GLU, and AST were measured. The results indicated serum TCHO and AST levels decreased in different dietary squalene supplementation groups, and GLU decreased in all groups. At the same time, the levels of TP, ALB, TBIL, and ALP increased to some extent in different groups of largemouth bass. The findings indicate that serum TCHO, GLU, and AST levels reduced in some squalene supplementation groups, which is consistent with previous studies. Administration of 1 g/kg squalene in rats’ diet for 4 weeks remarkably reduced blood pressure, blood glucose, TCHO, and TG levels, suggesting that squalene could modulate metabolic diseases such as cardiovascular disease by regulating sugar and lipid metabolism [61]. Elevated levels of TCHO and TG can result from excessive liver fat accumulation or lipid metabolism disorders. And it’s released into the bloodstream upon liver cell injury, leading to increased AST activity [28,60]. Therefore, in this study, the parameters of TCHO, GLU, and AST reduction in different groups indicate that squalene is beneficial to largemouth bass.

In fish, the liver is primarily responsible for fatty acid oxidation and lipid synthesis. Malondialdehyde (MDA), the end product of lipid peroxidation, serves as an indicator of the extent of oxidative damage induced by reactive oxygen species (ROS) [62]. Antioxidant enzymes such as superoxide dismutase (SOD) and catalase (CAT) scavenge excess ROS and mitigate oxidative damage [63]. The antioxidant properties of squalene in animals have been extensively investigated. For example, dietary squalene supplementation at 1–2 kg/tonne significantly reduced serum MDA levels and increased hepatic SOD activity in broilers [31]. A study on skin antioxidant function has demonstrated that various dosages of squalene significantly increased SOD and CAT activities in porcine skin [46]. In this experiment, dietary squalene supplementation significantly decreased hepatic MDA levels and increased SOD and CAT activities in the liver of largemouth bass, indicating that the squalene enhances antioxidant capacity in this species.

In addition, the intestinal tract is not only the primary site for nutrient digestion and absorption but also an immune organ in fish [64]. It secretes gastric acid, mucus, mucoproteins, bile, glycoproteins, mucopolysaccharides, and various digestive enzymes [65]. To a certain extent, digestive enzyme activity in the intestine reflects the fish’s digestive and absorptive capacity, as well as its ability to utilize dietary nutrients, which in turn affects growth and development. Therefore, digestive enzyme activity serves as a key parameter for evaluating diet efficacy in terms of growth promotion, feed intake, and nutrient utilization [66,67]. Digestive enzymes include trypsin, lipases, and amylase. Trypsin is an important animal-derived proteolytic enzyme that breaks down proteins into smaller amino acid peptides and amino acids [68]. Lipases are a group of relatively nonspecific fat-hydrolyzing enzymes capable of degrading various long-chain triglycerides [69]. Amylase primarily facilitates the digestion and absorption of starch, thereby promoting nutrient utilization and supporting healthy fish growth [70]. Previous studies have shown that squalene supplementation can improve digestive capacity and enhance intestinal structural integrity in grouper [33]. In weaned piglets, supplementation with 250 mg/kg squalene significantly increased jejunal villus height, reduced intestinal injury markers, improved jejunal antioxidant capacity, and alleviated early weaning stress-induced jejunal injury [50]. In the present study, the activities of trypsin, lipase, and α-amylase (AMS) in the intestine were markedly elevated in largemouth bass fed squalene-supplementation diets. To date, no studies have reported on the effects of squalene on intestinal digestive enzyme activity in largemouth bass. Our results indicate that squalene significantly improved hepatic antioxidant capacity, alleviated oxidative stress, and modulated lipid metabolism in this species, all of which positively influence growth and health. Furthermore, squalene supplementation was beneficial for liver and intestinal structure, as evidenced by histomorphological observations, such as compact, intact, and complete cellular organization, suggesting the capacity of squalene supplementation may enhance nutrient absorption capacity. Taken together, these findings provide a plausible explanation for the observed improvements in feed utilization and growth performance in largemouth bass following dietary squalene supplementation.

In this study, dietary squalene significantly improved hypoxic conditions in largemouth bass and prolonged breathing time under sealed conditions. It indicated that dietary squalene enhances the oxygen-carrying capacity of largemouth bass, thereby improving their tolerance to hypoxic conditions and increasing survival rates under low-oxygen stress. This characteristic is highly beneficial in situations of intensive farming, where there is a high density of animals and high oxygen consumption. It can improve breeding efficiency and reduce energy consumption. Previous studies have shown that squalene administration prolonged the breathing time of mice under normal baric hypoxia, sodium nitrite poisoning, and acute cerebral ischemic hypoxia [71]. Huang et al. reported that 30 days of squalene administration significantly increased survival time and breathing time in mice subjected to sodium nitrite injection and acute cerebral ischemic hypoxia [72]. It has been hypothesized that squalene possesses oxygen-carrying ability similar to that of red blood cells, enabling it to combine with oxygen in the body to form activated oxygenated squalene. This complex is then transported via the bloodstream to peripheral cells, where it releases oxygen, thereby promoting the biological redox reactions during metabolism and enhancing tolerance to hypoxia [42]. These results all confirm that squalene enhances oxygen-carrying capacity and hypoxia tolerance in animals.

As feed additives, plant extracts can not only improve growth performance but also enhance host immunity and disease resistance, and may even contribute to water quality regulation in aquaculture systems [73]. In the innate immune system, the first line of defense is mediated by *Mx*, a gene induced by interferons (IFNs) that inhibits the mid and late stages of the viral replication cycle. Fish, like mammals, exhibit robust induction of IFN-stimulated genes in response to viral infection, driven by *ifn* activation of a homologous gene cluster [74]. IRF3 (interferon regulatory factor 3) is a key regulator of type I IFN (IFN*-1*) transcription, and its activation correlates with IFN-1 induction. In fish, *ifn-γ* is classified as a type II *ifn* [75,76]. Beyond IFN-associated immune genes, oxidative stress is closely linked to immune responses in animals, and its persistence often leads to inflammation and various diseases [77,78]. Cytokine production, including both pro- and anti-inflammatory factors, serves as its primary regulator of the inflammatory response. Among these, IL-1β, IL-8, and TNF-α are key pro-inflammatory cytokines [79]. Consistent with the present findings, squalene supplementation has been shown to enhance disease resistance and immunity in grouper [33]. Studies in mice have demonstrated that squalene can downregulate the p38MAPK and NF-κB signaling pathways, thereby mitigating inflammatory responses [80]. Additionally, squalene may enhance immune function by inhibiting the synthesis and activity of xenobiotic-metabolizing enzymes, suppressing NF-κB and cyclooxygenase (COX) activity, and reducing the production of pro-inflammatory cytokines such as TNF-α [81]. In this study, dietary squalene supplementation in largemouth bass enhanced splenic expression of *mx*, *ifn-γ*, and *irf3* while decreasing expression of *il-1β*, *il-8*, and *tnf-α* compared with the control group. These results suggest that squalene modulates the innate immune system in largemouth bass, enhancing anti-inflammatory and anti-disease capabilities, and improving overall immune status and health. Furthermore, dietary squalene conferred protection against LMBRaV infection. Following LMBRaV infection, survival rates in squalene-supplemented groups were notably higher than those in the control group. This improved survival may be attributed to the upregulation of immune-related genes, which likely inhibit LMBRaV replication and infectivity.

Currently, intensive farming using artificial feed is the primary aquaculture model for largemouth bass, which imposes certain physiological pressure on the fish. Exposure to environmental stressors can lead to the accumulation of reactive oxygen species, impairing the immune and antioxidant systems and inducing cellular oxidative damage. This, in turn, increases susceptibility to disease and threatens the long-term sustainability of largemouth bass aquaculture. Therefore, effective regulation of lipid metabolism, enhancement of antioxidant capacity, mitigation of oxidative stress, and maintenance of overall fish health are crucial for improving disease resistance and supporting the healthy growth of largemouth bass.

## 5. Conclusions

This study demonstrated that dietary squalene supplementation in commercial feed for largemouth bass reduces serum blood lipids and glucose, promotes hepatic lipid metabolism, protects intestinal integrity, facilitates digestion and absorption, enhances hypoxia tolerance, and modulates immune-related gene expression, thereby exerting beneficial effects on growth performance and survival following LMBRaV infection. The optimal dietary supplement level for largemouth bass was 100 mg/kg. These findings highlight the great potential of squalene as a valuable dietary supplement in aquaculture.

## Figures and Tables

**Table 1:** Sequences of primer pairs used in this study.

Gene	Primer Sequences (5′-3′)	Reference/Accession No.
*β-actin*	F: CCACCACAGCCGAGAGGGAA R: TCATGGTGGATGGGGCCAGG	[54]
*mx*	F: TAAAATGGCTGGGGTCGGGG R: CATTGCACGGAACGACCACC	[55]
*ifn-γ*	F: AGATCAGAGGCTTTCAAATCCC R: CAACATGTGGCTAATCAGCTT	[54]
*irf3*	F: CCACTGCTCATCCCATGGCT R: GAGGAGTCCTGTCTTAAACC	XM_038735464.1
*tnf-α*	F: ACTTCGTCTACAGCCAGGCA R: AGTAACGCGAGACCCTGTGG	[55]
*il-8*	F: GAGTTTGAGGAGCCTGGGTGT R: GGGTCCAGGCAAACCTCTTG	[55]
*il-1β*	F: TGGTGGAAAACAGCATGGAGC R: AGGGTGCACGTAGTTCGACA	[55]

**Table 2:** Effects of squalene on the growth performance of largemouth bass.

Items			Groups		
	C	S1	S2	S3	S4
WGR (%)	94.97 ± 5.34 ^a^	113.08 ± 3.94 ^c^	112.30 ± 3.91 ^bc^	110.41 ± 4.68 ^bc^	99.81 ± 5.34 ^ab^
SGR (%/d)	2.38 ± 0.10 ^a^	2.70 ± 0.07 ^c^	2.69 ± 0.07 ^bc^	2.66 ± 0.08 ^bc^	2.47 ± 0.10 ^ab^
SR (%)	96.39 ± 0.35 ^c^	99.73 ± 0.46 ^d^	99.66 ± 0.58 ^d^	94.31 ± 0.85 ^b^	91.00 ± 1.00 ^a^
CF (g/cm^3^)	2.20 ± 0.13 ^a^	2.23 ± 0.12 ^a^	2.21 ± 0.12 ^a^	2.23 ± 0.18 ^a^	2.29 ± 0.17 ^a^
VSI (%)	7.82 ± 0.44 ^ab^	7.96 ± 0.32 ^b^	7.01 ± 0.45 ^a^	7.63 ± 0.68 ^ab^	7.36 ± 0.42 ^ab^
HSI (%)	2.12 ± 0.25 ^a^	2.23 ± 0.08 ^a^	2.14 ± 0.43 ^a^	2.04 ± 0.16 ^a^	1.80 ± 0.18 ^a^

Note: Values within the same row with different letters are significantly different (*p* < 0.05). WGR, weight gain rate, SGR: specific growth rate, SR, survival rate, CF: condition facto, viscerosomatic VSI: index, HSI: hepatosomatic index, C (control, 0 mg/kg squalene group), S1 (100 mg/kg squalene group), S2 (200 mg/kg squalene group), S3 (300 mg/kg squalene group), S4 (400 mg/kg squalene group).

## Data Availability

The data presented in this study are available within the article. Further inquiries may be directed to the corresponding authors.
